# In Vitro and In Vivo Evaluation of Starfish Bone-Derived β-Tricalcium Phosphate as a Bone Substitute Material

**DOI:** 10.3390/ma12111881

**Published:** 2019-06-11

**Authors:** Haruka Ishida, Hisao Haniu, Akari Takeuchi, Katsuya Ueda, Mahoko Sano, Manabu Tanaka, Takashi Takizawa, Atsushi Sobajima, Takayuki Kamanaka, Naoto Saito

**Affiliations:** 1 Institute for Biomedical Sciences, Interdisciplinary Cluster for Cutting Edge Research, Shinshu University, 3-1-1 Asahi, Matsumoto, Nagano 390-8621, Japan; 18hb401g@shinshu-u.ac.jp (H.I.); 19hb402j@shinshu-u.ac.jp (K.U.); 18bs212e@shinshu-u.ac.jp (M.S.); saitoko@shinshu-u.ac.jp (N.S.); 2 Department of Biomedical Engineering, Graduate School of Medicine, Science and Technology, Shinshu University, 3-1-1 Asahi, Matsumoto, Nagano 390-8621, Japan; taakari@shinshu-u.ac.jp; 3 Department of Biomedical Engineering, Graduate School of Science and Technology, Shinshu University, 3-1-1 Asahi, Matsumoto, Nagano 390-8621, Japan; 4 Department of Orthopaedic Surgery, Shinshu University School of Medicine, 3-1-1 Asahi, Matsumoto, Nagano 390-8621, Japan; m990054e@gmail.com (M.T.); takashitak@shinshu-u.ac.jp (T.T.); soba@shinshu-u.ac.jp (A.S.); kam17@shinshu-u.ac.jp (T.K.)

**Keywords:** starfish, calcium carbonate, porous calcium phosphate, β-tricalcium phosphate, bone substitute, angiogenesis

## Abstract

We evaluated starfish-derived β-tricalcium phosphate (Sf-TCP) obtained by phosphatization of starfish-bone-derived porous calcium carbonate as a potential bone substitute material. The Sf-TCP had a communicating pore structure with a pore size of approximately 10 μm. Although the porosity of Sf-TCP was similar to that of Cerasorb M (CM)—a commercially available β-TCP bone filler—the specific surface area was roughly three times larger than that of CM. Observation by scanning electron microscopy showed that pores communicated to the inside of the Sf-TCP. Cell growth tests showed that Sf-TCP improved cell proliferation compared with CM. Cells grown on Sf-TCP showed stretched filopodia and adhered; cells migrated both to the surface and into pores. In vivo, vigorous tissue invasion into pores was observed in Sf-TCP, and more fibrous tissue was observed for Sf-TCP than CM. Moreover, capillary formation into pores was observed for Sf-TCP. Thus, Sf-TCP showed excellent biocompatibility in vitro and more vigorous bone formation in vivo, indicating the possible applications of this material as a bone substitute. In addition, our findings suggested that mimicking the microstructure derived from whole organisms may facilitate the development of superior artificial bone.

## 1. Introduction

Bone is a tissue with excellent regenerative ability; however, supplementation is necessary for reconstruction of bone defects that cannot be naturally healed due to fracture or tumor resection. Autologous bone grafting can be used to compensate for defects. In this method, grafts are harvested from a healthy part of the patient and transplanted into the defective part of the bone. Because the graft itself has bone-forming ability, the process after transplantation is usually effective. However, it may not be possible to obtain bone of the necessary amount, shape, and size, and there are other disadvantages, such as pain and deformation at the collection site. Additionally, problems such as infection and immune response arise when allogeneic bone grafting and heterogeneous bone grafting are used [1].

In order to overcome these problems, researchers have been attempting to develop artificial bone prosthetic materials showing good biocompatibility [2]. For example, a glass material that bonds to bones without showing foreign body reactions was reported by Hench et al. in 1971 [3]. Since then, the development of artificial bone has progressed due to the discovery of physiologically active materials, even inorganic materials, and the synthesis of hydroxyapatite (HAp) as a main component of bone inorganic matter and direct bonding to bone were described by Jarcho et al. [4] and Akao et al. [5]. In addition to HAp, β-tricalcium phosphate (β-TCP) has been extensively studied as a calcium phosphate-based biomaterial. β-TCP has higher solubility at neutral pH than HAp and is not only hydrolyzed in body fluids but is also biologically absorbed by osteoclasts, representing an effective bioabsorbable material that can be used as a bone substitute. Studies on β-TCP have been reported by Driskell et al. [6], and SynthoGraft, which was first developed in 1981, has been approved by the US Food and Drug Administration as an absorbable synthetic bone grafting material. Such bioceramics account for approximately 40% of total bone grafts [7].

Regardless of material, porous bodies with continuous pores are required for supporting osteoinductive factors and for binding, after implantation, with strong bone, cellular tissues, and blood vessels for invasion [8]. It is necessary to control pore size distribution and pore structure in order to achieve both secured pore structures that promote bone formation and strength when used as a filler material. Porous bodies have been produced by various methods [9,10]. However, no artificial bone with performance comparable to bone has been developed.

Roy and Linnehan have reported methods for converting coral-derived calcium carbonate into HAp as an artificial bone using biological materials [11]. Coral-derived HAp is a porous HAp with a pore size of 150 to 500 μm and has high biocompatibility [12]. This material has been commercialized as ProOsteon in the United States of America. However, coral harvesting is costly and associated with environmental problems.

The inorganic matter forming on starfish is composed of calcite granules containing Mg^2+^ and has a fine porous structure with a pore diameter of several tens of microns. Takeuchi et al. reported that this porous structure is converted into β-TCP containing Mg^2+^ by phosphatization of starfish [13].

Therefore, in this study, we evaluated the physical properties of starfish bone-derived β-TCP as a potential bone substitute material using MC3T3-E1 mouse calvaria-derived osteoblast-like cells and in vivo implantation into rat calvaria bone defects.

## 2. Materials and Methods 

### 2.1. Ethics Statement

All experiments were carried out according to institutional guidelines for animal experimentation at Shinshu University School of Medicine. All protocols used in this study were reviewed and approved by the Division of Laboratory Animal Research (#280054). All surgery was performed under general anesthesia (intraperitoneal injection of sodium pentobarbital), and all efforts were made to minimize suffering. All rats were euthanized using isoflurane inhalation at the end of the study.

### 2.2. Preparation of Porous Calcium Phosphate Derived from Starfish Bone

Collection of starfish-bone-derived calcite and its phosphatization to β-TCP were carried out as previously described [13]. Organic substances were dissolved by immersing starfish (*Patiria pectinifera*) in a commercial bleach (sodium hypochlorite aqueous solution with a volume ratio of about 6%; Hiter^®^; Kao Corp., Tokyo, Japan). After dissolving organic matter, starfish bone was obtained by filtering, washing with ion-exchanged water, and drying at 60 °C for 24 h. Briefly, 1.5 g of starfish bone granules were phosphatized in a reaction vessel for hydrothermal treatment (inner volume 25 mL) with 20 mL of a 0.5 mol/L diammonium hydrogen phosphate aqueous solution [(NH_4_)_2_HPO_4_, FUJIFILM Wako Pure Chemical Corp., Osaka, Japan], at 200 °C for 72 h. Phosphatization treatment was performed under more severe conditions than autoclave sterilization, so samples were considered sterile and treated accordingly. The product was then washed with ion-exchanged water and dried at 60 °C for 24 h.

The obtained phosphate-treated starfish-bone-derived β-TCP (Sf-TCP) was sieved at 150–500 μm to obtain materials with the same size as the β-TCP used in the clinical setting (Cerasorb M [CM]; curasan AG, Kleinostheim, Germany). 

### 2.3. Observation of Surface Structures by Scanning Electron Microscopy (SEM)

The Sf-TCP was affixed on a brass sample holder using conductive carbon paste (DOTITE XC-12, JEOL Ltd., Tokyo, Japan), coated with osmium oxide (FUJIFILM Wako Pure Chemical Corp.) by an osmium coater (Neoc-AN; Meiwafosis, Tokyo, Japan) at 10 mA for 20 s, and then observed under a field emission-scanning electron microscope (FE-SEM, JSM-7600F; JEOL Ltd., Tokyo, Japan) at an accelerating voltage of 2.00 kV.

### 2.4. Measurement of Specific Surface Area

The specific surface area of Sf-TCP was measured in triplicate with a high-precision multisample gas adsorption amount measuring device (Autosorb^®^-iQ; Quantachrome Instruments, Kanagawa, Japan) using the nitrogen adsorption method. Before measuring, vacuum degassing was performed at 120 °C for 3 h. Specific surface area was calculated from the range of relative pressures of adsorption-desorption isotherms by 0.05 to 0.35 according to the BET theory formula, as follows:

Monomolecular adsorption:

(1) $$\upsilon_{m}=\upsilon \left(1-\frac{p}{p_{0}}\right)$$

ν:Adsorption amount

$\frac{p}{p_{0}}:\mathrm{Relative}\;\mathrm{pressure}$

Surface area:

(2) $$A_{s}=\left(\frac{\nu_{m}{\mathrm{Na}}_{m}}{m}\right)\times 10^{-18}$$

N:Avogadro constant 

$a_{m}:\mathrm{Molecular}\;\mathrm{occupied}\;\mathrm{cross}\;\mathrm{section}\;$

m:Molecular weight of adsorbate

### 2.5. Measurement of Porosity and the Most Frequent Pore Diameter

We then used MicrotracBEL (Osaka, Japan) to measure porosity and the most frequent pore diameter of Sf-TCP by the mercury intrusion method using an automatic mercury porosimeter (Pascal Model 140; Thermo Scientific, Tokyo, Japan). Density was measured by the helium gas replacement method using a true density measuring apparatus (BELPycno, MicrotracBEL, Osaka, Japan). Before measuring, vacuum degassing was performed at room temperature for 15 min. Each item was measured once. The measured samples were used for subsequent experiments.

### 2.6. Cell Growth Tests

Mouse calvaria-derived osteoblast-like cells, MC3T3-E1 (RIKEN Cell Bank, Tsukuba, Japan) were used as cultured cells. MC3T3-E1 cells were cultured in an incubator at 37 °C in an atmosphere containing 5% CO_2_. αMEM medium supplemented with 10% fetal bovine serum and 1% antibiotic-antimycotic mixed solution was used for cell culture. For passaging of cells, cells were seeded into 10 cm dishes at a density of 4.0 × 10^4^ cells/mL and subcultured twice a week.

For cell proliferation tests, Alamar Blue assay (Alamar Blue Cell Viability Reagent, Remel, Lenexa, KS, USA) was used. MC3T3-E1 cells were seeded into 96-well plates at 6.0 × 10^3^ cells/well. After culturing for 24 h, medium was exchanged and used for experiments.

Sf-TCP and CM were added at 10 mg/well. The control wells contained cells only. As a sample blank, evaluation targets were added to medium and wells without cells were used. Those groups consisted of eight wells, each with two sample blanks. 

After addition of evaluation targets, the cells were cultured for 24 h. For the Alamar Blue assay, Alamar Blue Cell Viability Reagent was added to 10% of the total volume and reacted for 1 h. The Alamar Blue assay was performed by reading the fluorescence intensity at an excitation wavelength of 535 nm and an emission wavelength of 590 nm using a Plate Reader (AF2200, Eppendorf, Hamburg, Germany).

### 2.7. Cell Observation on the Sf-TCP Surface

The Sf-TCP cultured with MC3T3-E1 cells for 24 h was removed with tweezers, fixed by freeze-drying in 2.5% glutaraldehyde (used after dilution of 70% glutaraldehyde, TAAB Laboratories Equipment, Berks, UK), 1% osmium solution (aqueous solution of osmic acid, Nisshin EM, Tokyo, Japan), and t-butyl alcohol (FUJIFILM Wako Pure Chemical Corp.) and observed under an FE-SEM at an accelerating voltage of 15.0 kV.

### 2.8. Implantation in a Rat Calvaria Defect Model

Experimental animals were male Wistar rats (8 weeks old, weighing 150–200 g, SLC, Hamamatsu, Japan). According to animal experiment guidelines, rats were housed at five rats per cage in a breeding room with controlled room temperature (25 °C ± 2 °C) and humidity (50% ± 10%). Food and water were available ad libitum.

In accordance with the methods described by Tanaka et al. [14], a rat calvaria defect model was established. Briefly, after induction of anesthesia by isoflurane (Forane, ABBOTT JAPAN, Tokyo, Japan) inhalation, pentobarbital (Somnopentyl, Kyoritsu Seiyaku, Tokyo, Japan) was subcutaneously injected at 40 mg/kg, and the operation was performed. Bone defects of 5 mm in diameter were made in the rat calvaria using a trephine bar. No implant was placed in the sham (i.e., control) group, whereas the experimental groups were implanted with 10 mg Sf-TCP or CM. Rats were then housed for 4 or 8 weeks. There were eight rats in each group. Rats that died during the 4–8 week period were not used for evaluation.

### 2.9. Histological Examination

After euthanasia under anesthesia, the heads of rats were dissected and fixed for 1 week with 10% formalin (FUJIFILM Wako Pure Chemical Corp.). Rat skulls were then decalcified for 3 days inquick dehydrating liquid (K-CX, Pharma, Tokyo, Japan). Samples after demineralization were processed to an appropriate size and embedded in paraffin. After embedding in paraffin, samples were sliced into sections (4 μm thick) using a microtome. Sections were then subjected to hematoxylin (Muto Pure Chemicals, Tokyo, Japan) and eosin (FUJIFILM Wako Pure Chemical Corp.) staining (HE staining) and Masson’s Trichrome (MT) staining (Muto Pure Chemicals) and observed with an optical microscope (BX50, Olympus, Tokyo, Japan).

MT-stained specimens were observed with a multispectral automatic tissue section quantitative analysis system (Vectra3, PerkinElmer, Waltham, MA, USA) and photographed as multispectral images. Upon quantification, dye used for MT staining was incorporated and analyzed with inForm (PerkinElmer). For each specimen, we selected and photographed the inForm analysis part manually such that the area occupied by CM and Sf-TCP was maximized within the photograph range of 250 μm × 334 μm defined by Vectra3. The threshold for quantitative analysis was set automatically. Analysis results quantified by pixel number were averaged for each group and calculated for the area of fibrous tissue and cells in the bone defect area.

### 2.10. Statistical Analysis

For statistical analysis, Student’s t-tests were used, and for multiple comparisons, Bonferroni corrections were performed. Statistical results were expressed as means ± standard errors (SEs). The significance level was set at *p* < 0.05.

## 3. Results

### 3.1. Size of CM and Sf-TCP

**Figure 1:**
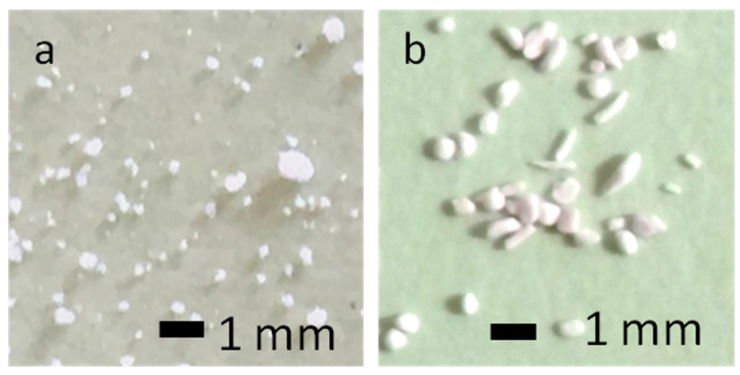
Photographs of the analyzed materials. (**a**) Cerasorb M (CM). (**b**) starfish-derived β-tricalcium phosphate (Sf-TCP) after sieving.

Figure 1 shows photographs of CM, as shown in Figure 1a, and Sf-TCP, as shown in Figure 1b, after sieving according to the catalog size of CM. The catalog CM size was 150–500 μm, but some particles of less than 150 µm were present.

### 3.2. Surface Structure

**Figure 2:**
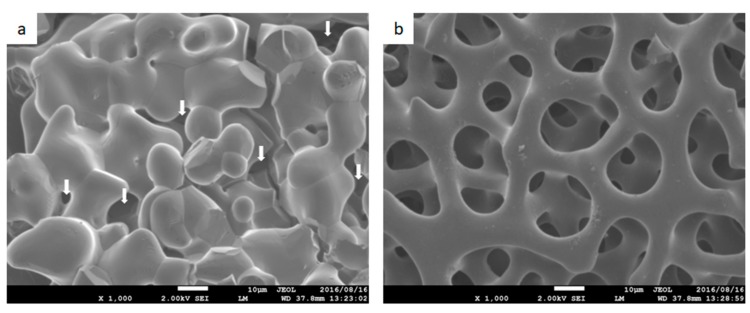
Surface images obtained by scanning electron microscopy. Magnification: ×1000. (**a**) CM. Arrows indicate pores. (**b**) Sf-TCP. Scale bar: 10 µm.

Figure 2 shows ×1000 SEM images of CM, as shown in Figure 2a, and Sf-TCP, as shown in Figure 2b. In CM, pores of various shapes were observed on the surface, as shown by the arrows in Figure 2a. In Sf-TCP, pores were found all over the surface, and the structures were connected with other pores inside.

### 3.3. Density, Most Frequent Pore Diameter, and Porosity

Table 1 shows measurement results of density and porosity. The density of the material was approximately 3 g/cm^3^. The total pore volume and total pore surface area for Sf-TCP were 445.42 mm^3^/g and 0.130 m^2^/g, respectively, whereas those for CM were 415.31 mm^3^/g and 0.062 m^2^/g, respectively. The Sf-TCP pore surface area was approximately twice that of CM. CM had a median pore diameter of about 57 μm and a most frequent pore diameter of about 100 μm, representing a 2-fold difference. For Sf-TCP, the median diameter and most frequent pore diameter were about 10–12 μm, which showed almost no difference. Additionally, pores that excluded mercury were observed in Sf-TCP in porosity measurements by mercury intrusion.

### 3.4. Specific Surface Area

**Figure 3:**
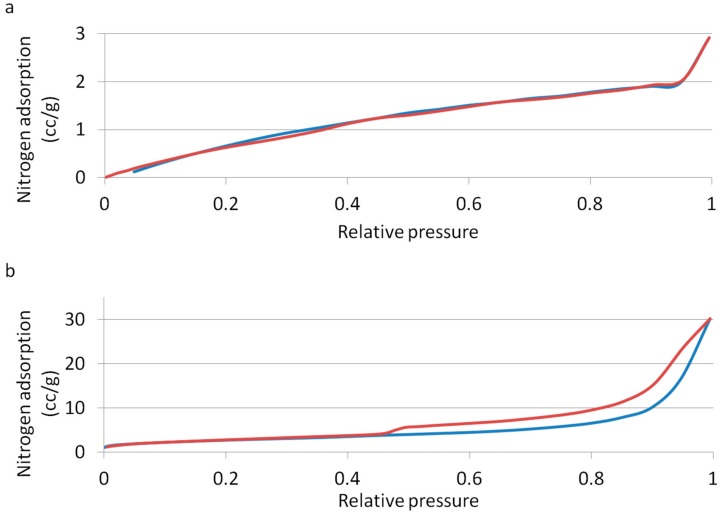
Adsorption-desorption isotherms. The blue line indicates adsorption, and the red line indicates desorption. (**a**) CM; (**b**) Sf-TCP.

Figure 3 shows the adsorption-desorption isotherm resulting from measurement of CM, as shown in Figure 3a, and Sf-TCP, as shown in Figure 3b, using the nitrogen adsorption method. 

The calculated specific surface area of CM was 3.693 m^2^/g, and that of Sf-TCP was 9.676 m^2^/g; thus, Sf-TCP was about three times larger than CM.

From the shape and hysteresis of the materials with a relative pressure range of adsorption-desorption isotherms between 0.45 and 1.00, the pore structure of the surface could be identified by IUPAC classifications [15]. CM was found to be a type II material, having macropores with a diameter of 50 nm or more or no pores, whereas Sf-TCP was a type V material having mesopores with a diameter of 2–50 nm.

### 3.5. Cell Growth Test

**Figure 4:**
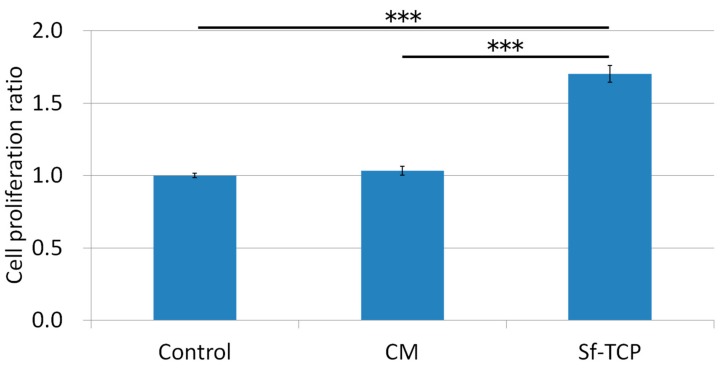
Effects of CM and Sf-TCP on cell proliferation. Values are the means ± standard errors (SEs) (*n* = 6). ***P < 0.001.

Figure 4 shows the results of the Alamar Blue assay as a ratio of the control when 10 mg Sf-TCP or CM was added. The positive control CM showed no significant differences compared with the control group. In contrast, Sf-TCP-treated cells showed more than a 1.5-fold increase compared with the control and CM groups.

### 3.6. Observation of Cells on the Sf-TCP Surface

**Figure 5:**
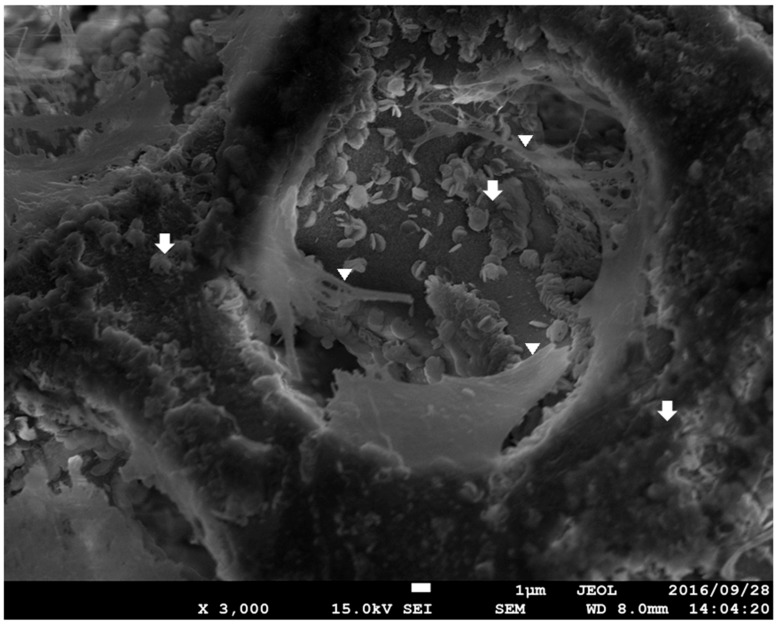
Surface SEM image of Sf-TCP cultured with MC3T3-E1 cells. Magnification: ×3000. The arrows show the scaly structure on the surface, and the triangles show cell filopodia.

Figure 5 shows ×3000 SEM images of Sf-TCP after culture with MC3T3-E1 cells. A scaly microstructure was observed on the Sf-TCP surface. MC3T3-E1 cells adhered not only to the Sf-TCP skeleton but also to pores with stretched filopodia.

### 3.7. Histological Examination of HE-Stained Specimens 

**Figure 6:**
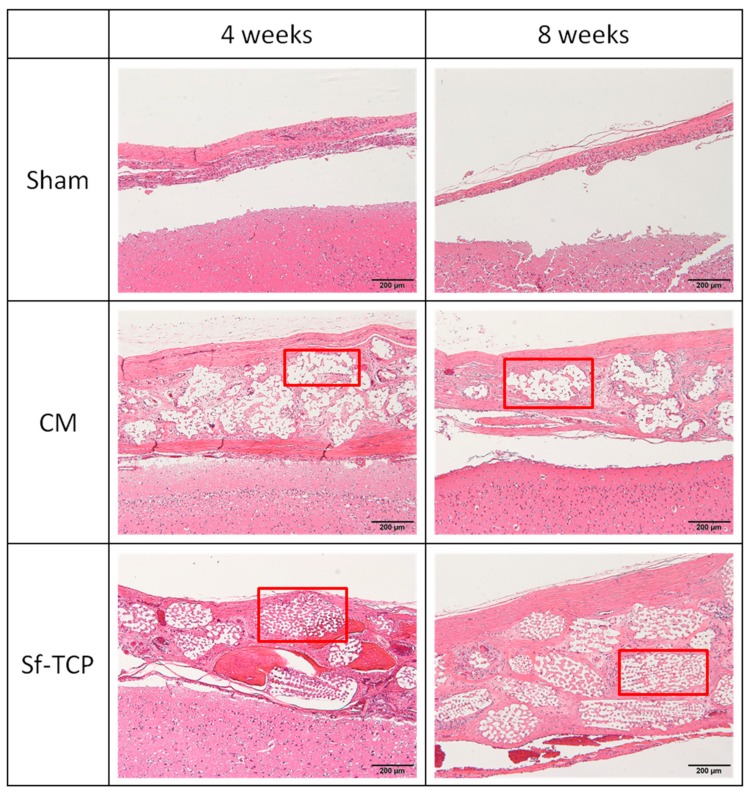
Hematoxylin and eosin (HE) staining. Hematoxylin stains cell nuclei blue, while eosin stains the cytoplasm, connective tissue, and other extracellular substances pink or red. Brain tissues are shown in the lower portion of the images, and the outer side of the skull is shown in the upper portion of the images. Boxed parts are prosthetic materials. Scale bar: 200 μm.

Figure 6 shows photographs of HE-stained specimens. In the sham group, there was a thin tissue layer covering the bone defect. In contrast, in the CM group, the amount of tissue present in CM was nearly unchanged from 4 to 8 weeks. In the Sf-TCP group, tissue was already present in the porous structure of Sf-TCP at 4 weeks.

**Figure 7:**
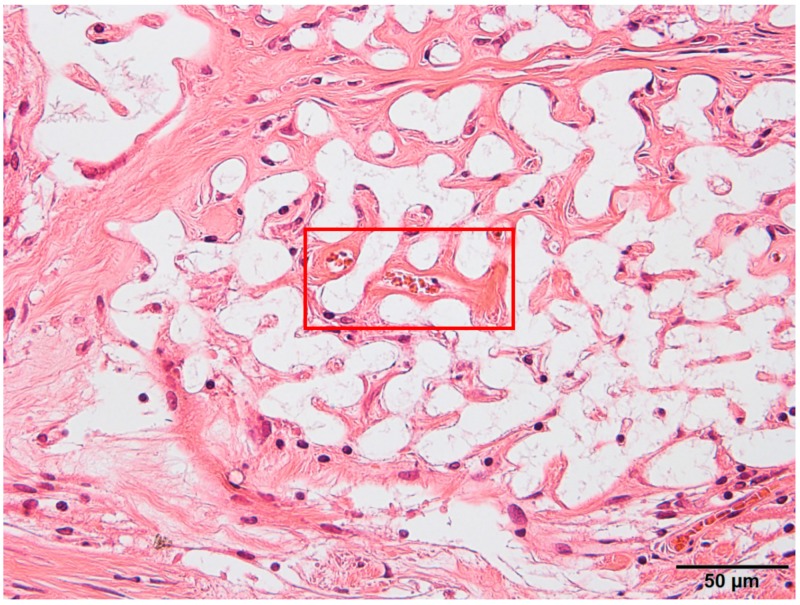
Blood vessel structures (red boxed area) were identified by hematoxylin and eosin (HE) staining.

Notably, vascular structures were observed at 8 weeks after implantation in the Sf-TCP group, as shown in Figure 7. Capillary structures were observed only in the Sf-TCP group.

### 3.8. Observation and Quantification of MT-Stained Specimens 

**Figure 8:**
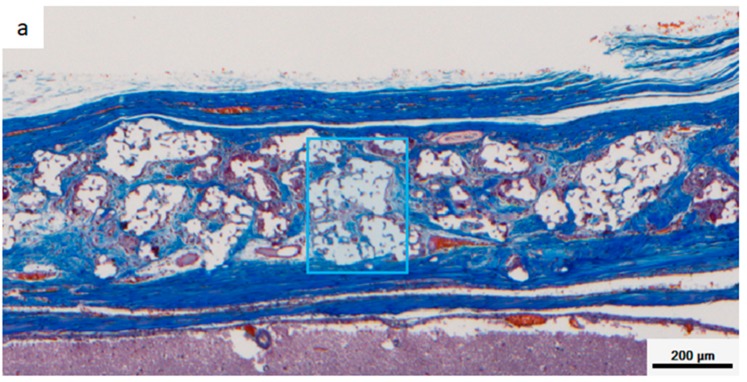

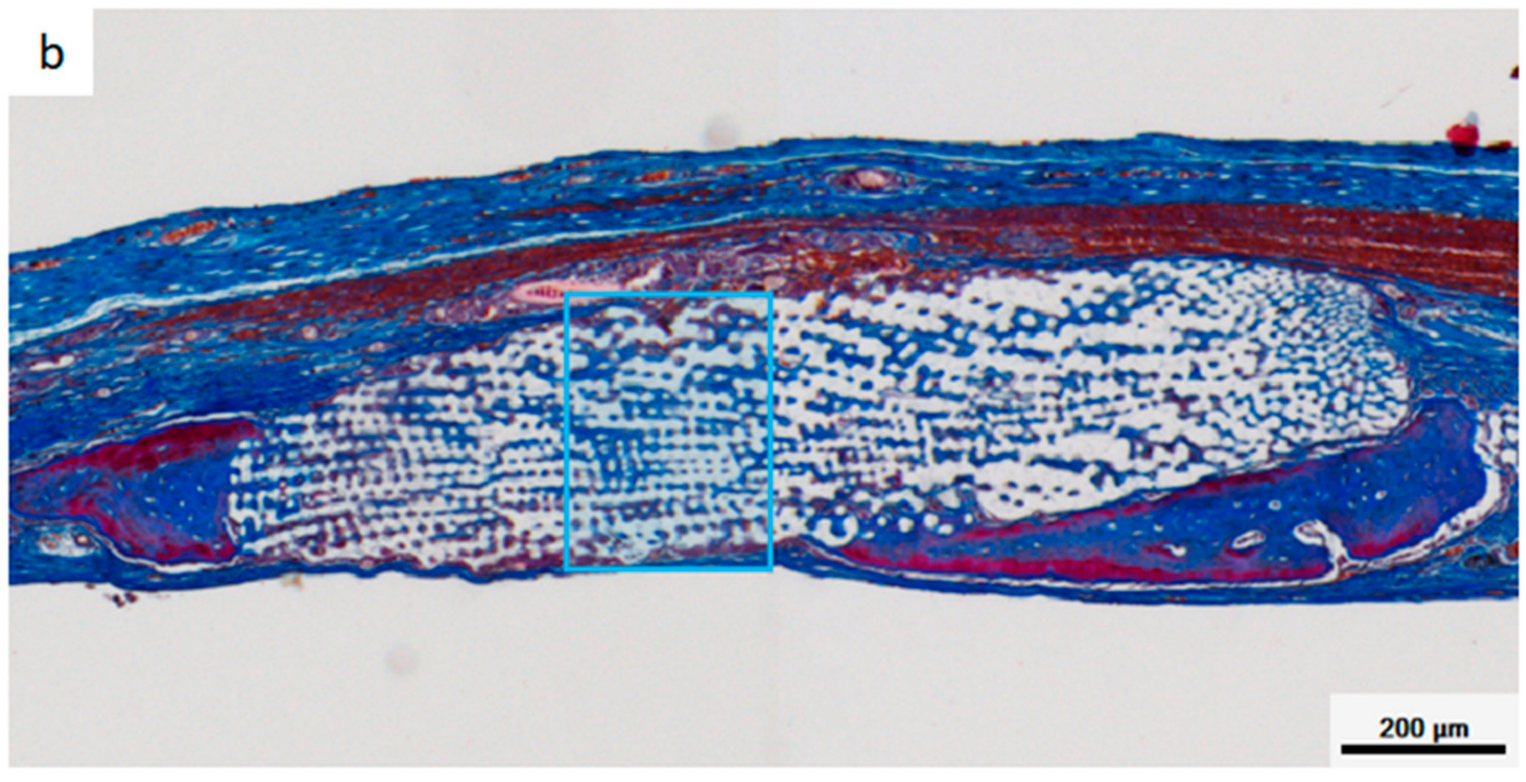
Masson’s trichrome (MT)-stained images taken with Vectra3. Aniline blue stains fibrous tissue blue, Masson liquid stains cells red, and Orange G stains blood cells orange. (**a**) CM implant at 4 weeks. (**b**) Sf-TCP implant at 4 weeks. The blue boxes indicate the area quantified in Figure 10.

Figure 8 shows a multispectral image of MT-stained specimens in the CM and Sf-TCP groups. Aniline blue stains fibrous tissue blue, Masson liquid stains cells red, and Orange G stains blood cells orange. For quantitative analysis with inForm, CM and Sf-TCP existed in the bone defects.

**Figure 9:**
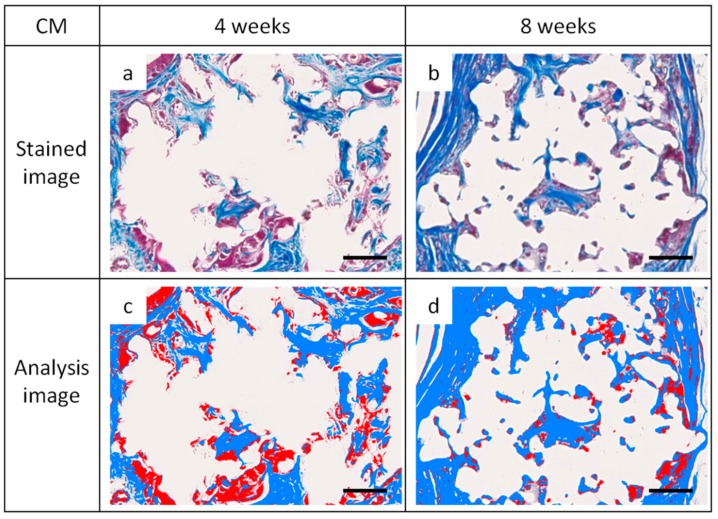

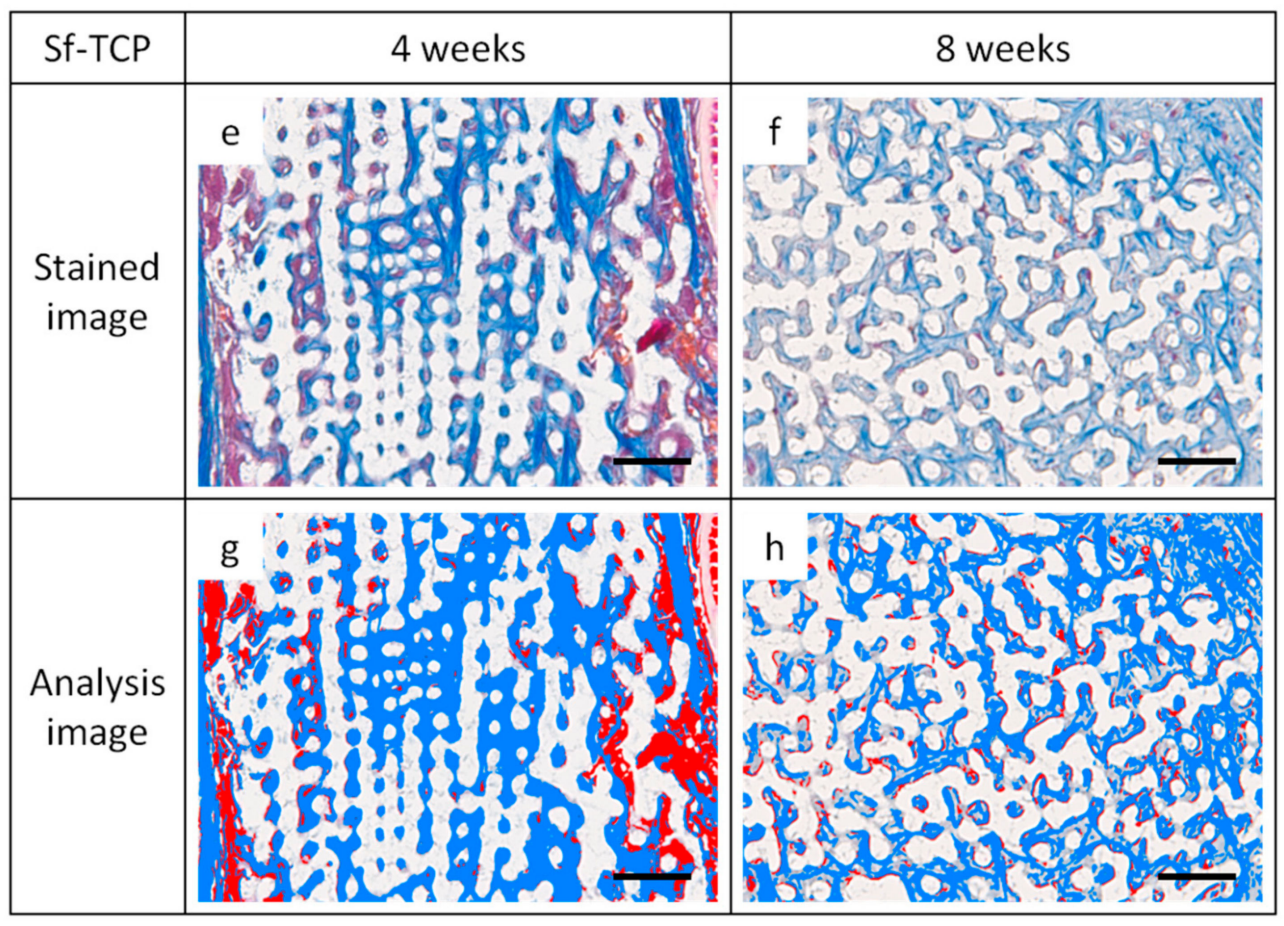
Images of Masson’s trichrome (MT)-stained sections and analysis images for quantification. Representative images showing cells and fibrous tissue existing mainly around CM or Sf-TCP pores. Cells appear more uniformly scattered at 8 weeks than at 4 weeks. (**a**,**b**) CM images with MT staining. (**c**,**d**) CM images for analysis. (**e**,**f**) Sf-TCP images with MT staining. (**g**,**h**) Sf-TCP images for analysis. Aniline blue staining (blue) shows fibrous tissue, and Masson liquid and Orange G staining (red) show cells. Scale bar: 50 μm.

Figure 9 shows an enlarged image of the region selected in Figure 8 and an image obtained by analyzing the corresponding portion with inForm. Cells and fibrous substances existed mainly in the periphery of CM in the CM group at 4 and 8 weeks, whereas in the Sf-TCP group, cells and fibers entered into the pores of Sf-TCP.

**Figure 10:**
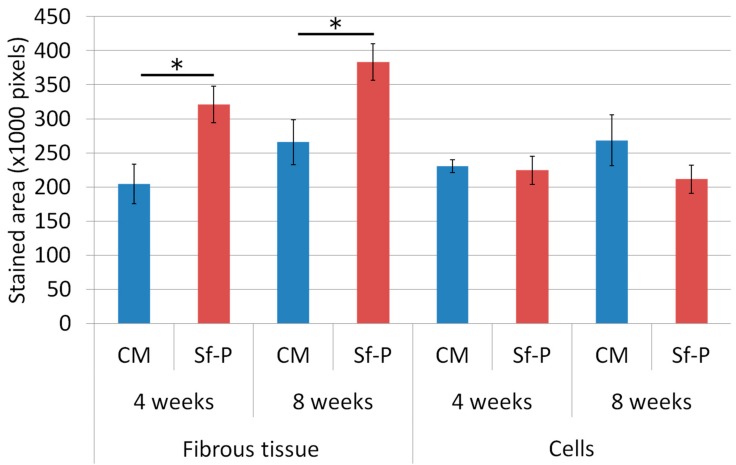
Quantitative analysis using inForm. The numbers of samples used for analysis were five in the Sf-TCP group at 4 weeks and six in other groups. Mean ± SEs. * *P* < 0.05.

Figure 10 shows the results of quantifying areas of fibrous tissues and cells. Fiber and cell areas in the CM and Sf-TCP groups were compared at 4 and 8 weeks. The results showed that the fiber mass was significantly larger in the Sf-TCP group than in the CM group at both 4 and 8 weeks. However, actual cell amount did not change significantly.

**Figure 11:**
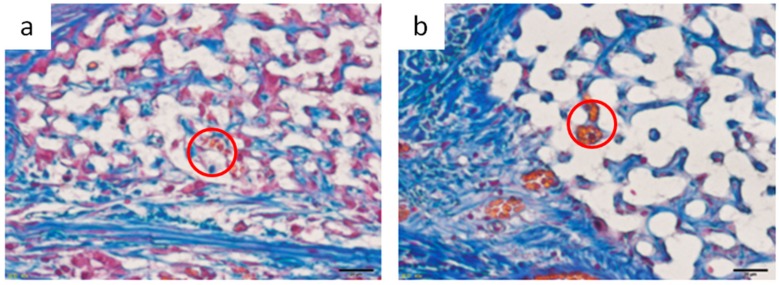
Image of erythrocytes observed inside Sf-TCP. (**a**) 4 weeks. (**b**) 8 weeks. Red circles indicate erythrocytes observed in Sf-TCP. Scale bar: 20 μm.

Figure 11 shows images of erythrocytes stained with Orange G inside the implant at the time of observation, and Table 2 shows the number of individuals exhibiting erythrocytes in the prosthetic material. These results were observed only in the Sf-TCP group (one of five animals at 4 weeks and three of six animals at 8 weeks).

## 4. Discussion

In this study, we evaluated the physical properties of Sf-TCP and performed in vitro and in vivo experiments to examine the potential applications of Sf-TCP as a bone substitute material. Our results showed that St-TCP did not induce cytotoxicity, which is important for the development of biomaterials, and facilitated the healing of wounds in vivo, supporting the potential clinical applications of this material.

Our results showed that Sf-TCP induced significant improvement in cell proliferation compared with the control or CM treatments. These findings were thought to be related to the excellent cell adhesion and proliferation properties of Sf-TCP. Indeed, the specific surface area of Sf-TCP was about three times larger than that of CM, consistent with studies demonstrating that cell proliferation increases as the specific surface area increases [16,17]. Because cell proliferation is a main factor indicating biocompatibility in MC3T3-E1 cells [18], our results supported that Sf-TCP was highly biocompatible. Additionally, in morphological observations, MC3T3-E1 cells adhering to Sf-TCP showed well-stretched filopodia, which has been reported to occur during cell migration [19], and the structure of Sf-TCP indicated that the cells could migrate.

In our in vivo experiments, cells and fibrous tissues invaded into the communicating porous structures of Sf-TCP in MT-stained specimens, indicating that cellular tissues could penetrate Sf-TCP, even when the pores were 10–12 μm in size. In addition, increased fibrous tissue area was observed in the Sf-TCP group compared with that in the CM group. The amount of fibrous material has been reported to be related to the abundance of collagen fibers and calcification [20,21]. Thus, the large amount of fibers present in Sf-TCP may indicate progression of bone formation. In porous artificial bone prosthetic materials, new bone invades into the pores and binds to surrounding bone [11]; accordingly, our findings supported the performance of Sf-TCP as a bone substitute material.

In the Sf-TCP group, vascular structures were observed in HE-stained specimens, and erythrocytes were present inside Sf-TCP in MT-stained specimens, suggesting the formation of capillary vessels. The pore size of Sf-TCP (10–12 μm) was smaller than that of previously reported biomaterials (100 μm), which allows invasion by cells and tissues, or that (300 μm) required for angiogenesis, e.g., invasion of capillaries [22]. However, the pore sizes of HAp and β-TCP are typically within the range of 100 to 600 μm (or 150 to 200 μm when coral is used as a biological material) [23,24,25,26]. Therefore, it is possible that few studies have evaluated such small pores or that angiogenesis was not observed in CMs, which have pores of 50–100 μm because of differences between artificial and biologically-derived porous structures. Various factors, such as disconnection of communicability by closed pores, variations in pore size, and sharp connecting structures with respect to the size of cells in artificial communication pores, are thought to hinder invasion of cellular tissues. However, in Sf-TCP, there were few of these structures and few elements that inhibited the invasion of cellular tissues; thus, vigorous tissue invasion and formation of capillary vessels would have occurred. In the rat calvaria defect model, capillary vessels are approximately 4–10 μm in diameter in the cerebrum around the defect, and capillaries can invade pores with a diameter of 10–12 μm. This result is important in considering the three-dimensional structure of the potential prosthetic bone filling material. Moreover, artificial preparation of starfish bone-like structures adapted to the capillary diameters of humans may yield artificial bone prosthetic materials capable of invading capillaries and tissues while improving the strength of the material by decreasing the pore size. Additionally, pore sizes increase when β-TCP is dissolved, absorbed, and replaced with autologous bone, and tissues larger than the pore may therefore invade the material.

## 5. Conclusions

In this study, we evaluated whether Sf-TCP derived from starfish could be used as a bone substitute material. Compared with CM, which is already used clinically, Sf-TCP was found to have comparable porosity but a larger specific surface area. Cell proliferation tests showed that Sf-TCP promoted cell proliferation. Moreover, in animal experiments, Sf-TCP showed high regeneration ability, vigorous invasion of cellular tissue into the pore structure, and introduction of capillary vessels into the prosthetic material, even when the pore size was only approximately 10 μm. However, this study did not examine the pore size required for angiogenesis, so there may be a more appropriate pore size. In the future, we hope to optimize regeneration accompanied by angiogenesis by artificially creating materials with structures similar to that of Sf-TCP but with different pore diameters.

From the above results, we concluded that Sf-TCP obtained by phosphatization of starfish bone may be effective for bone regeneration applications, such as in the treatment of fractures and bone loss. In addition, mimicking the structure of the starfish bone may lead to the development of superior artificial bone substitute materials.

## Figures and Tables

**Table 1:** Density, most frequent pore diameter, and porosity.

Sample Name	CM	Sf-TCP
Sample weight (g)	0.1960	0.2061
Density (g/cm^3^)	3.0665	2.9158
Total pore volume (mm^3^/g)	415.31	445.42
Total pore surface area (m^2^/g)	0.062	0.130
Median pore diameter (µm)	57.86	12.37
Most frequent pore diameter (µm)	99.78	10.22
Porosity (mercury penetration was possible) (%)	56.42	52.44
Porosity (mercury penetration was not possible) (%)	−0.72	7.18
Total porosity (%)	55.70	59.62

**Table 2:** Number of individuals having erythrocytes in prosthetic material (number/total).

Material	4 Weeks	8 Weeks
CM	0/6	0/6
Sf-TCP	1/5	3/6
